# Comparative analysis of clinical features of lower respiratory tract infection with respiratory syncytial virus and influenza virus in adults: a retrospective study

**DOI:** 10.1186/s12890-023-02648-5

**Published:** 2023-09-15

**Authors:** Jiahua Tian, Congyue Liu, Xunling Wang, Ling Zhang, Guoying Zhong, Guichuan Huang, Hongping Wang, Hao Hu, Ling Gong, Daishun Liu

**Affiliations:** 1 Zunyi Medical University, Zunyi, 563000 China; 2 Institute of Chinese Medical Sciences, University of Macau, Taipa, Macau SAR China; 3 Department of Respiratory Medicine, The First People’s Hospital of Zunyi (The Third Affiliated Hospital of Zunyi Medical University), Zunyi, 563000 China

**Keywords:** Clinical characteristic, Comparison and analysis, Influenza virus, Lower respiratory tract infection, Respiratory syncytial virus

## Abstract

**Background:**

Respiratory syncytial virus (RSV) infection in adults remains less recognized and understood, both socially and clinically, compared to influenza virus infection. This retrospective study aims to delineate and compare the clinical manifestations of adult RSV and influenza virus infections in the lower respiratory tract, thereby enhancing awareness of RSV lower respiratory tract infection and providing strategic insights for its prevention and treatment.

**Methods:**

Clinical data from January 2019 to December 2020 were analyzed for 74 patients with RSV and 129 patients with influenza A/B virus lower respiratory tract infections who were admitted to respiratory or intensive care units. All patients had complete clinical data with positive IgM and negative IgG viral antibodies. Comparison parameters included onset timing, baseline data, clinical manifestations, supplementary examination results, treatment methods, and prognosis, while logistic regression was employed to ascertain the correlation of clinical features between the two patient groups.

**Results:**

In comparison to the influenza group, the RSV group presented less frequently with fever at admission but exhibited a higher incidence of dyspnea and wheezing on pulmonary auscultation (*P* < 0.01). RSV infection was more prevalent among patients with underlying diseases, particularly chronic obstructive pulmonary disease (COPD) and demonstrated a higher probability of co-infections, most notably with Mycoplasma (*P* < 0.01). The RSV group had significantly higher lymphocyte counts (*P* < 0.01) and exhibited more incidences of pleural thickening, pulmonary fibrosis, and emphysema (*P* < 0.05). The use of non-invasive mechanical ventilation was more common, and hospital stays were longer in the RSV group compared to the influenza group (*P* < 0.05). Logistic multivariate regression analysis further revealed that age and tachypnea incidence were significantly higher in the RSV group (*P* < 0.05).

**Conclusion:**

Compared to influenza virus infection, adults with COPD are more susceptible to RSV infection. Moreover, RSV infection elevates the risk of co-infection with Mycoplasma and may lead to conditions such as pleural thickening, pulmonary fibrosis, and emphysema. The requirement for non-invasive mechanical ventilation is higher in RSV-infected patients, who also tend to have longer hospital stays. Therefore, greater awareness and preventive strategies against RSV infection are imperative.

## Introduction

Acute respiratory disease, primarily due to acute viral respiratory infections, significantly contributes to global morbidity and mortality, with influenza virus being the most recognized pathogen. However, respiratory syncytial virus (RSV) often receives less attention despite ranking third in respiratory viral pathogen detection [1–3]. Globally, RSV accounts for 94,600 to 149,400 annual deaths in children under five, and an estimated 336,000 adult hospitalizations, with approximately 14,000 in-hospital fatalities [3–7]. These statistics underscore the crucial need to acknowledge the significant clinical burden of RSV infection.

Both RSV and influenza predominantly present as upper respiratory tract infections. Risk factors for progression to lower respiratory tract infections may include age, immunocompromise, and pre-existing conditions [8–10]. The rising RSV infection rate, coupled with its comparable hospital mortality rate to influenza, underscores RSV’s considerable contribution to human disease burden [11]. In certain adults, especially those who are immunocompromised, elderly, or with cardiopulmonary disease, RSV can lead to life-threatening exacerbations of bronchial asthma or COPD [12, 13]. Notably, 10–31% of adult RSV patients require intensive care, 3–17% require mechanical ventilation, and mortality can reach 6–8% [11]. These alarming figures underline the urgency to increase the focus on RSV infection.

Thus, this study provides a retrospective analysis of 74 adult hospitalized patients with RSV IgM antibody positive and IgG antibody negative and 129 adult hospitalized patients with influenza A/B virus IgM antibody positive and IgG antibody negative, all presenting with lower respiratory tract infection from January 2019 to December 2020. By comparing the clinical characteristics, this study aims to enhance public understanding of RSV’s role in adult lower respiratory tract diseases, providing valuable insights for clinical prevention and treatment of RSV infection.

## Methods

### Participants and study design

The participants in this study were adults diagnosed with lower respiratory tract infections due to either RSV or influenza virus in the Respiratory Department and the Comprehensive ICU at the First People’s Hospital of Zunyi (The Third Affiliated Hospital of Zunyi Medical University) from January 2019 to December 2020.

The inclusion criteria were: (1) age 18 years and older; (2) presence of respiratory virus IgM antibodies but absence of IgG antibodies; and (3) fulfillment of European diagnostic standards for lower respiratory tract infections [11]. Exclusion criteria were: (1) age under 18 years; (2) upper respiratory tract infections; (3) diagnosis of lower respiratory tract infection more than 48 h following admission; (4) long-term chemotherapy or hormone use or incomplete clinical data; and (5) incapacity to cooperate due to neuropsychiatric disorders. Prior to study commencement, all participants provided written informed consent [14].

Before the start of the study, we will contact all patients included in this retrospective study to obtain their consent and sign the written informed consent. The study received ethical approval from the Ethics Committee of the First People’s Hospital of Zunyi City (Third Affiliated Hospital of Zunyi Medical University) (Approval No. 2020-049, dated October 2020), adhering to the Helsinki Declaration principles of 1964.

### IgM and IgG antibody detection of nine respiratory pathogens

Blood samples used in this study were primarily obtained from fasting, hospitalized adults within the initial 24 h of admission. Experienced nursing staff collected these samples at 6 am, verifying patient identity and location per standardized protocols before informing them about the purpose and procedure of the blood collection. A sterile, single-use venipuncture needle was connected to a disposable venous blood sample collection container following hand sanitation. Following elbow disinfection with iodine, 2–3 mL of venous blood was collected. Subsequent to the withdrawal of the needle, patients were guided to apply pressure to the site of venipuncture with a sterile cotton swab. Patient identity and location were verified once more, and the sample container was appropriately labelled. The collected samples were immediately transported to the laboratory for IgM and IgG antibody testing. Trained laboratory personnel conducted an indirect immunofluorescence assay (IFA) to detect antibodies against nine respiratory pathogens, namely: *Mycoplasma pneumoniae*, influenza B virus, respiratory syncytial virus, parainfluenza virus, influenza A virus, adenovirus, *Chlamydia pneumoniae*, *Legionella pneumophila*, and *Coxiella burnetii*. The respiratory infection detection kit used was sourced from Zhengzhou Antu Bioengineering Co., Ltd. The imaging analysis was performed using the OLYMPUSBX-51 fluorescence microscope imaging system, a product of Olympus Corporation, Japan.

### Data collection

A retrospective review of patient medical records was conducted, and the following information was recorded: age, gender, underlying conditions, clinical features (e.g., cough, sputum, tachypnea, fever, wheezing, wet rale), laboratory test results (e.g., white blood cell, neutrophil, monocyte, lymphocyte and platelet counts, aspartate aminotransferase, alanine aminotransferase, creatine kinase, creatine kinase isoenzyme, lactic dehydrogenase levels), chest CT scans, sputum bacteriological cultures, ICU admission status, implementation of invasive or non-invasive mechanical ventilation, length of hospital stay, and inpatient mortality rates.

### Statistical analysis

Statistical analysis was performed using SPSS 25.0. Quantitative data not normally distributed was represented using the interquartile range, with the rank sum test employed for comparisons. Normally distributed measurement data was expressed as (x ± s) and evaluated with the *t*-test. Categorical data was reported as [example (%)] and analyzed using the χ^2^ test. Logistic regression analysis was conducted on selected indicators displaying statistical differences, with *p* values < 0.05 deemed statistically significant.

## Results

### Prevalence and seasonality of RSV and influenza virus

**Fig. 1:**
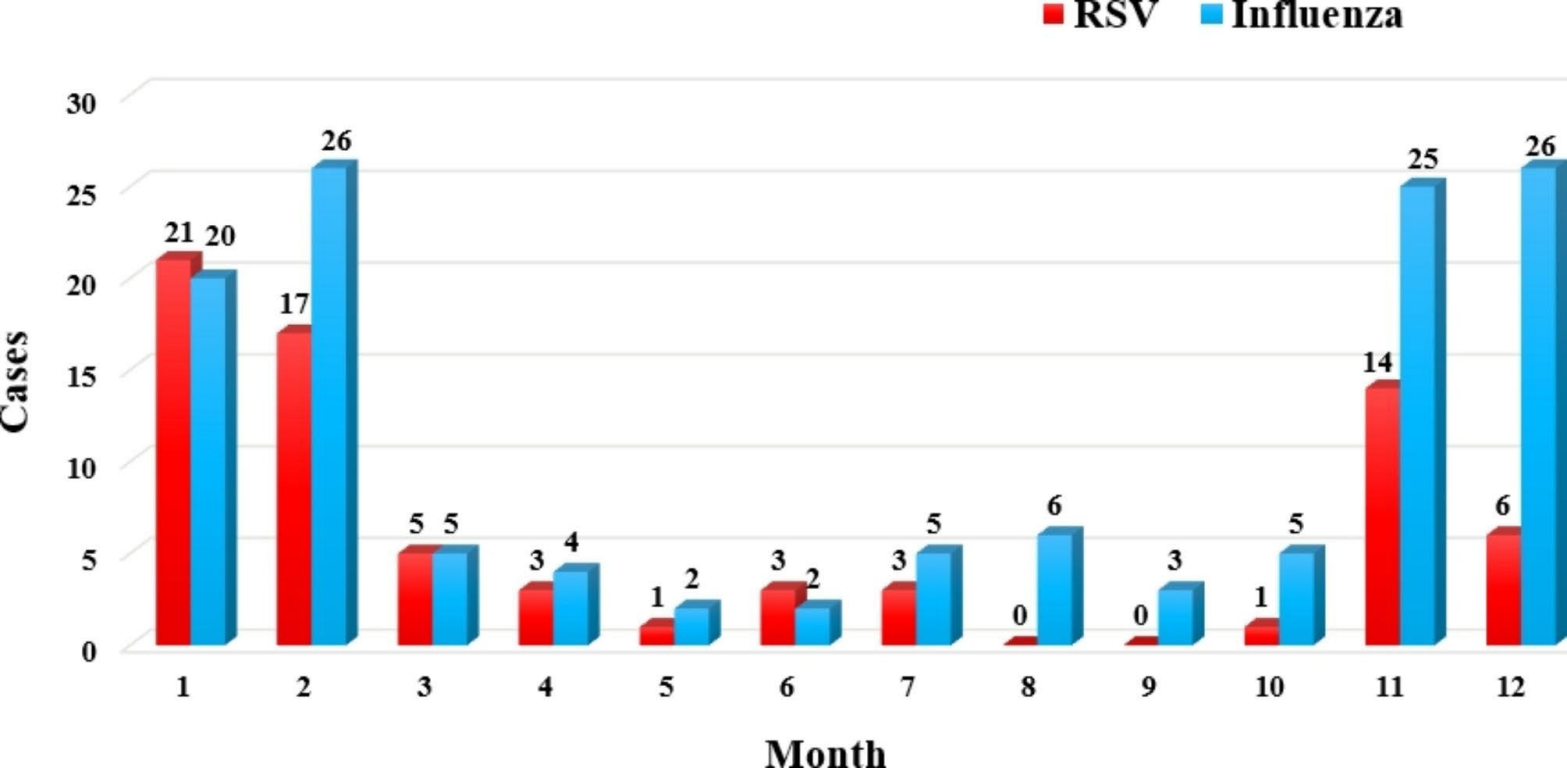
Temporal distribution of RSV and influenza cases in patients with lower respiratory tract infection

The incidence of influenza virus infection surpassed that of RSV during the two-year study period. The epidemic peak of both viruses was commonly observed in the winter months of January-February and November-December, accounting for 62% and 75% of total RSV and influenza cases, respectively. A significant decrease in infection rates for both RSV and influenza was observed during the non-peak months (Fig. 1).

### Demographic and clinical characteristics of RSV and influenza patients

Among the cohort of 74 patients diagnosed with RSV infection, the average age was 65.5 years, significantly higher than the 58-year mean age of the 129 influenza virus patients (*P* < 0.05). The RSV group comprised 32 males and 42 females, possessing an average age of 61.14 ± 19.28 years, while the influenza cohort included 49 males and 80 females, with a mean age of 54.64 ± 18.14 years. No significant difference in the age distribution was observed between the genders within either group (*P* > 0.05). Comorbidities were present in approximately 73% of the RSV patients, with 30 (40.50%) demonstrating chronic obstructive pulmonary disease (COPD), 24 (32.40%) having heart disease, 12 (16.20%) with diabetes, 12 (16.20%) with bronchiectasis, and 4 (5.40%) with bronchial asthma. The prevalence of underlying conditions, particularly COPD, was significantly higher in the RSV group compared to the influenza cohort (*P* < 0.01). No significant disparity was observed regarding the other comorbidities, namely bronchial asthma, bronchiectasis, diabetes, and heart disease, between the two groups (*P* > 0.05) (Table 1).

**Table 1:** Baseline data of RSV and influenza virus patients with lower respiratory tract infection

	RSV infection group (N = 74)	Influenza virus infection group (N = 129)	Z/χ²/*t*	*p* value
General item				
Age	65.5 (46.5, 77)	58 (44, 69)	-2.524	0.012*
Gender (e.g., %)				
Male	32 (43.20)	49 (38.00)	0.542	0.461
Female	42 (56.80)	80 (62.00)		
Underlying disease (ex., %)				
Underlying disease	54 (73)	62 (48.10)	11.916	0.001**
COPD	30 (40.50)	25 (19.40)	10.660	0.001**
Bronchial asthma	4 (5.40)	3 (2.30)	0.574	0.449
Bronchiectasis	12 (16.20)	10 (7.80)	3.487	0.062
Diabetes	9 (12.20)	15 (11.60)	0.013	0.910
Heart disease	24 (32.40)	31 (24.00)	1.680	0.195

Statistically significant, *P < 0.05, **P < 0.01

### Clinical and auxiliary examination characteristics of patients with RSV and influenza virus lower respiratory tract infection

RSV and influenza virus infections presented similar primary clinical symptoms: cough and sputum production. Secondary symptoms, in order of prevalence, were pulmonary auscultation findings of wet rales, dyspnea, wheezing, followed by fever. However, the secondary symptom ranking differed for influenza virus infection, with fever taking precedence, followed by wet rales on auscultation, dyspnea, and wheezing. Relative to influenza patients, RSV patients exhibited fewer instances of fever but reported increased dyspnea and wheezing upon pulmonary auscultation, with these differences reaching statistical significance (*P* < 0.01). Additionally, RSV patients demonstrated a higher likelihood of concurrent infections, particularly with Mycoplasma, a difference that was statistically significant (*P* < 0.01). In contrast, the prevalence of bacterial and other viral co-infections showed no significant disparity (*P* > 0.05) (Table 2).

Upon admission, RSV patients had significantly elevated lymphocyte counts compared to influenza patients (*P* < 0.01). However, there were no statistically significant differences in the counts of white blood cells, neutrophils, monocytes, or platelets, nor in the levels of ALT, AST, CK, CK-MB, and LDH (*P* > 0.05). Radiological findings revealed lung consolidation, pleural thickening, pulmonary fibrosis, emphysema, bilateral pneumonia, and pleural effusion in both patient groups. Yet, RSV patients were more likely to present pleural thickening, pulmonary fibrosis, and emphysema (*P* < 0.05, *P* < 0.01, *P* < 0.05 respectively) (Table 2).

**Table 2:** Clinical features and auxiliary examination characteristics of RSV and influenza virus patients with lower respiratory tract infection

	RSV infection group (N = 74)	Influenza virus infection group (N = 129)	Z/χ²/*t*	*p* value
Symptoms and signs (e.g., %)				
Cough	61 (82.40)	99 (76.70)	0.911	0.340
Expectoration	53 (71.60)	88 (68.20)	0.257	0.612
Shortness of breath	25 (33.80)	14 (10.90)	15.932	<0.001**
Fever	22 (29.70)	75 (58.10)	15.212	<0.001**
Wheezing sound	22 (29.70)	7 (5.40)	22.683	<0.001**
Wet rales	36 (48.60)	47 (36.40)	2.903	0.088
Co-infection (ex., %)				
Have co-infection	64 (86.50)	79 (61.20)	12.773	<0.001**
Bacteria	28 (37.80)	39 (30.20)	1.230	0.267
Mycoplasma	44 (59.50)	32 (24.80)	24.110	<0.001**
Other viruses	10 (13.50)	23 (17.80)	0.643	0.422
Laboratory examination				
White blood cells (10^9^/L)	7.9 (5.65, 9.90)	7.59 (5.52, 10.20)	-0.671	0.502
Neutrophils (10^9^/L)	6.20 (4.37, 8.72)	5.68 (3.81, 8.03)	-1.113	0.266
Monocytes (10^9^/L)	0.62 (0.35, 0.79)	0.48 (0.33, 0.73)	-1.824	0.068
Lymphocytes (10^9^/L)	1.19 (0.94, 1.64)	0.85 (0.58, 1.40)	-3.684	<0.001**
Platelets (10^9^/L)	187.5 (125.25, 242.00)	192 (140.00, 240.50)	-0.252	0.801
ALT (ummol/L)	20.50 (12.00, 33.25)	20 (12.00, 33.00)	-0.194	0.846
AST (ummol/L)	26 (19.75, 37.25)	26 (18.50, 39.50)	-0.322	0.748
CK (U/L)	64.50 (44.75, 94.25)	74 (46.50, 120.00)	-1.395	0.163
CK-MB (U/L)	13.50 (10.75, 18.00)	13 (10.00, 17.00)	-0.325	0.745
LDH (U/L)	232.5 (196.50, 292.00)	225 (188.00, 314.50)	-0.670	0.503
Chest CT (ex., %)				
Lung consolidation	1 (1.40)	4 (3.10)	0.092	0.761
Pleural thickening	9 (12.20)	5 (3.90)	5.028	0.025*
Pulmonary fibrosis	22 (29.70)	6 (4.70)	24.873	<0.001**
Emphysema	22 (29.70)	20 (15.50)	5.800	0.016*
Double pneumonic infiltration	43 (58.10)	76 (58.90)	0.013	0.911
Pleural effusion	24 (32.40)	35(27.10)	0.641	0.423

Statistically significant, *P < 0.05, **P < 0.01

### Treatment and prognosis of RSV and influenza virus patients with lower respiratory tract infection

With respect to management and prognostic outcomes, non-invasive mechanical ventilation was predominantly observed in RSV-infected patients, and the duration of hospitalization was more extended compared to those with an influenza virus infection (*P* < 0.05 and *P* < 0.01, respectively). However, both groups exhibited no significant differences in terms of ICU admissions, the necessity for invasive mechanical ventilation, and in-hospital mortality rates (*P* > 0.05) (Table 3).

**Table 3:** Treatment and prognosis of RSV and influenza virus patients with lower respiratory tract infection

	RSV infection group (N = 74)	Influenza virus infection group (N = 129)	Z/χ²/t	*p* value
Treatment and prognosis (case, %)				
ICU admission	13 (17.60)	17 (13.20)	0.719	0.396
Invasive mechanical ventilation	8 (10.80)	19 (14.70)	0.626	0.429
Non-invasive mechanical ventilation	17 (23.00)	14 (10.90)	5.339	0.021*
Length of stay (number of days)	10 (6.75, 15.25)	8 (4.00, 12.50)	-3.515	<0.001**
In-hospital fatality rate	1 (1.40)	2 (1.60)	0.000	1.000

Statistically significant, *P < 0.05, **P < 0.01

### Univariate and multivariate logistic regression analysis

In order to elucidate the associations between the clinical manifestations of RSV and influenza virus infections, parameters such as age, bilateral pneumonic infiltration, LDH, CK-MB, CK, ALT, AST, platelet count, lymphocyte count, monocyte count, existence of coexisting diseases, dyspnea, and mechanical ventilation were incorporated into univariate logistic regression analysis.

The results showed statistical significant differences in age (*P* < 0.05), concurrent infection, lymphocyte count, presence of comorbid conditions, tachypnea, and the requirement for mechanical ventilation (all *P* < 0.01) (Table 4).

**Table 4:** Univariate analysis of clinical characteristics of RSV and influenza virus cases with lower respiratory tract infection

Variables	β	Wald	OR	95% CI	*p* value
Age	0.019	5.515	1.020	1.003–1.036	0.019*
Double pneumonic infiltration	-0.033	0.013	0.967	0.542–1.727	0.911
co-infection	1.399	13.197	4.015	1.904–8.616	<0.001**
LDH	-0.001	0.477	0.999	0.997–1.001	0.490
CK-MB	0.017	0.992	1.018	0.983–1.053	0.319
CK	-0.001	0.893	0.999	0.996–1.001	0.345
ALT	0.000	0.003	1.000	0.994–1.005	0.958
AST	0.000	0.015	1.000	0.995–1.005	0.903
Platelets	0.000	0.039	1.000	0.996–1.003	0.843
Lymphocyte count	0.605	9.227	1.916	1.260–2.915	0.002**
Monocyte absolute value	0.546	1.527	1.726	0.724–4.115	0.218
Co-underlying disease	1.071	11.516	2.918	1.572–5.416	0.001**
Shortness of breath	1.433	14.611	4.191	2.010–8.738	<0.001**
Mechanical ventilation	1.085	9.329	2.960	1.475–5.939	0.002**

Statistically significant, *P < 0.05, **P < 0.01

Further logistic multivariate regression analysis demonstrated that the incidence of age and shortness of breath was higher than that of influenza virus-infected patients (*P* < 0.05, *P* < 0.01, respectively) (Table 5).

**Table 5:** Multifactor analysis of clinical characteristics in RSV and influenza virus cases with lower respiratory tract infection

Variables	β	Wald	OR	95% CI	*p* value
Age	0.020	4.238	1.021	1.001–1.041	0.040*
Shortness of breath	1.323	10.110	3.753	1.661–8.481	0.001**

Statistically significant, *P < 0.05, **P < 0.01

## Discussion

Historically, there has been a disproportionate focus on influenza virus infection, with both patients and clinicians frequently underestimating the escalating morbidity and mortality associated with RSV infection in adults. Despite the higher prevalence and ease of diagnosis of influenza, emerging data suggest that outcomes may be poorer for patients with RSV infection [15–17]. This study provides an improved understanding of the incidence and clinical manifestation of RSV lower respiratory tract infection in adults, thereby enhancing both societal and clinical recognition of this disease.

Our study found that RSV infection peaks during the November to February period, essentially paralleling the epidemic season of influenza. This concurs with findings by Nam et al. [3], who similarly noted that RSV infection peaks in the winter months, specifically December and January.

Assessment of baseline demographics revealed that RSV infection is more common among slightly older individuals compared to those with influenza, which could be attributed to age-related declines in memory T cell immune function [3]. Furthermore, patients with existing chronic pulmonary disease or major comorbidities are more susceptible to RSV infection than influenza [18, 19]. Hämäläinen et al. [20] and Coussement et al. [11] independently found a higher incidence of RSV infection among older patients with an elevated disease burden, particularly those with chronic respiratory conditions. To corroborate this, our team conducted a preliminary investigation in which we detected RSV infection in 20% of the sputum samples randomly collected from 20 AECOPD patients, [21] underscoring the increased vulnerability of this patient population to RSV infection.

In this investigation, we identified cough and sputum as the most prevalent symptoms among both RSV-infected and influenza virus-infected patients. RSV-infected patients exhibited lower frequencies of fever compared to their influenza counterparts. However, these patients reported higher incidences of dyspnea and wheezing as detected via lung auscultation. Despite these variations in symptomology, they are insufficient to conclusively differentiate between RSV and influenza virus infections. Moreover, our study identified a predisposition for RSV lower respiratory tract infections to coincide with other pathogenic infections. Existing literature posits that this mechanism of co-infection may be attributable to respiratory epithelial cell barrier damage, alterations in airway function, heightened susceptibility to additional pathogens, overexpression of adhesion proteins, and irregularities in the host immune response [22, 23]. Prior research indicates that co-infections are more prevalent with bacteria such as *Streptococcus pneumoniae* and *Haemophilus influenzae* in RSV patients [24]. Nevertheless, our findings suggest a greater likelihood of Mycoplasma co-infection in RSV patients, possibly due to regional variations or diverse study populations. In terms of laboratory results, this study identified higher lymphocyte counts upon admission in RSV patients compared to those with influenza virus infection. Literature shows a correlation between influenza virus infection and lymphocyte depletion, which may be a result of lymphocyte migration to lung lesions during infection [25]. The exact mechanism behind this, however, necessitates further clinical observation and documentation. Regarding radiological findings, lung consolidation, pleural thickening, pulmonary fibrosis, emphysema, bilateral pneumonia infiltration, and pleural effusion were identified in both patient groups. However, the likelihood of pleural thickening, pulmonary fibrosis, and emphysema appeared to be heightened in RSV patients compared to those with influenza virus infection. There is no existing literature on this topic, indicating the need for further clinical analysis and documentation.

Regarding therapeutic interventions and prognosis, RSV patients demonstrated a higher propensity for requiring mechanical ventilation, albeit without significant differences in in-hospital mortality compared to influenza patients. Our results are congruent with the findings by Coussement et al. [11], which revealed a higher incidence of mechanical ventilation in RSV patients but similar in-hospital mortality rates for both groups. In contrast, Matias et al. [26] reported lower in-hospital fatality rates for RSV compared to influenza, suggesting variations depending on the severity of the respective infections. These findings underscore the potential burden of ICU admissions for RSV infection in adults and stress the need for effective preventative measures and early intervention in the treatment of RSV infection [16]. Univariate regression analysis in our study revealed age, co-infection, lymphocyte count, concurrent underlying conditions, dyspnea, and mechanical ventilation to be more significantly correlated with RSV infection than with influenza virus infection. Moreover, multivariate regression analysis demonstrated that age and dyspnea have a more substantial correlation with RSV infection compared to influenza virus infection.

Serum IgM is an indicator for diagnosis in acute infection phase and also plays an important role in the clinical diagnosis and treatment of virus infection. Due to its high sensitivity, reverse transcription-polymerase chain reaction (RT-PCR) has become the gold standard for respiratory virus test. However, it is difficult to determine whether the pathogen is associated with lower respiratory diseases, and there is a certain number of false negatives in the diagnosis of some infected cases. Serological testing (IgM and IgG) can support RT- PCR to provide a more accurate assessment of infectivity. A study about the molecular detection of respiratory viruses in hospitalized patients with community-acquired pneumonia reported that RT-PCR and serological analysis had good consistency in detecting RSV infection, and serological detection significantly improved the diagnostic rate of virus infection [27].

Despite the often-underestimated significance of RSV infection in clinical practice, our research underscores its potential for severe outcomes, particularly when coupled with co-infections. This realization necessitates the reprioritization of RSV infection in medical management, including active surveillance, and the appropriate therapeutic interventions when required. Prophylactically, the vaccination of patients with COPD and the elderly population, especially during high transmission seasons, is advised. Furthermore, RSV testing should be a standard component of pathogen detection protocols for patients exhibiting respiratory infection symptoms. Once an RSV infection is confirmed, treatment regimens may need to incorporate monoclonal antibodies, depending on the illness’s severity, to mitigate complications, reduce hospitalization duration, and lower intensive care unit admission rates.

Nonetheless, the present study acknowledges certain limitations. The diagnostic scope was confined to serum antibody IgM and IgG testing, lacking comprehensive nucleic acid data of respiratory pathogens. Furthermore, the study’s single-center nature limits generalizability, warranting further multi-center validation. Lastly, the relatively small sample size for both RSV and influenza virus infections necessitates future studies with larger cohorts to permit more robust analysis.

## Conclusion

This study highlights the heightened risk posed by RSV infection over influenza virus infection to adults with COPD, including an increased propensity for Mycoplasma co-infection and the subsequent development of complications such as pleural thickening, pulmonary fibrosis, and emphysema. Additionally, RSV infection has been linked to an increased demand for non-invasive mechanical ventilation and prolonged hospitalization. Therefore, a reemphasis on preventing and managing RSV infection is of utmost importance, given its substantial clinical implications for COPD patients.

## Data Availability

All data generated or analyzed during this study are included in this published article.

## Declarations

049, date October 2020).

## Abbreviations

COPD: Chronic obstructive pulmonary disease
RSV: Respiratory syncytial virus
ALT: Alanine aminotransferase
AST: Aspartate aminotransferase
CK: Creatine kinase
CK-MB: Creatine kinase isoenzyme
LDH: Lactate dehydrogenase
ICU: Intensive care unit
CI: Confidence interval
β: Regression coefficient
wald: Chi-square value
OR: Odds ratio
